# To a Hopeless Dyspeptic

**Published:** 1890-03

**Authors:** 


### TO A HOPELESS DYSPEPTIC.

Bad as you are, you are still young, and if you will take our advice, we will cure you, and not only cure you, but give you a stomach like that of an ostrich. The first thing you have to do is to consider that what is called modern cookery is a conspiracy against human health. Among simple people living on a few things, it would not be necessary to take the precautions that you will have to take if you want to get well. First of all, get into your head a list of things that are bad for you and tend to produce the state in which you now are.

WHAT TO AVOID.

Alcohol in every form.
Beverages (effervescing).
Biscuits.
Bread (bakers' and fermented).
Butchers' meat (in quantity).
Butter.
Cakes.
Cheese.
Coffee.
Confectionery (every sort).
Creams.
Curries.
Dried meats.
Duck.
Dumplings.
Eels.
Fish (oily).
Fruits (acid and dried).
Goose.
Grease.
Herring.
Honey.
Ices.
Jams.
Jellies.
Lard.
Lemons.
Liquid food (in every form).
Mackerel.
Made dishes.
Malt liquors.
Marmalade.
Medicines (of every kind).
Meats. salted, dried, smoked, and otherwise preserved.
Milk.
Nuts.
Oil.
Pastry (baked or boiled).
Pickles.
Pies.
Pills (of every kind).
Pork.
Preserved meats.
Puddings.
Rhubarb.
Salads (dressed).
Salmon.
Salted meats.
Sauces.
Sausages.
Smoked meats.
Soups.
Spices.
Suet.
Sugar (and all foods containing it).
Tea.
Tinned things.
Tobacco in every form.
Tonics (of every kind).
Veal.
Very hot food.
Vinegar.
Wines.

### WHAT TO EAT.

Every fool, and, above all things, the untravelled fool, will probably laugh at this, and tell you there is nothing left to eat; but that would be a great mistake. One cf the mistakes of the untravelled person, and particularly the cockney, is that he does not appear to have the slightest idea that whole peoples—some of the best of peoples—live on one or two simple things exclusively. You have several important classes of food.

*Cereals.*—Rice, oatmeal, barley, wheat, and its many products, like maccaroni (which is a much better food than bread) and vermicelli, hominy and tapioca.

*Vegetables.*—Many delicate things, from asparagus to spinach, the best being the green vegetables; roots like turnips, carrots, and parsnips are not quite so easily digested nor so good for delicate people as the green vegetables like French beans, young peas, and asparagus.

*Fruits* of Europe, America, and the Tropics, which are introduced in abundance. For delicate people some fruits are perhaps too rich—like some pears and bananas—but others are most delicate and wholesome, especially eaten as food raw or simply stewed.

*Fish.*—In our country plentiful, and most excellent food—any of the white kinds of fish, like sole, turbot, whiting, pollock, and brill. Oily fish, such as salmon, mackerel, herrings, and eels, should be avoided. Fish should always be eaten fresh, and grilled, roasted, or boiled—roast fish is excellent, so is grilled.

*Game.*—The game of our country, and, indeed, of all countries, is for dyspeptic people infinitely preferable to butchers' meat, for the reason which we cannot detail here, but which are sound. Nothing can be more wholesome than roasted or broiled game.

*Poultry.*—Except ducks and geese. So here you see. without going to *butcher's meat* at all, there are many different kinds of food. If mutton or beef are eaten by a dyspeptic person they should be cooked simply and eaten in very small quantities.

You will observe that, milk puddings being forbidden, rice must be taken in the proper way—as a vegetable (in which way it is excellent) and together with stewed fruits of various kinds. It can be used admirably without being made into heavy milk puddings as it often is; so of similar foods like maccaroni, which in England is mainly used as a pudding, whereas its true use is a simple and excellent plain food.

As you may have doubts how to combine these foods we will tell you of ways that we know to be perfectly successful.

*Breakfast about* 8.—The best of all things for breakfast is well-boiled oatmeal porridge, never boiled for less than an hour and a quarter—better an hour and a half; also brown, unfermented bread, and fruit in season. No bakers' bread should be eaten, and no very coarse brown bread, because that is irritating to some people. If you want more, there is broiled fish or broiled game.

*Lunch about* 1.—For a sedentary man lunch ought to be of one or two light things, such as bread (of the right kind) or stewed fruit with plain rice or other cereal, or a little plain mutton broth, without grease, and a little bread; or, in cases where cookery cannot be had, a couple of such a good apple as the Newtown pippin, and a piece of plain bread; or a sandwich of game or fowl, or a sandwich of fresh meat

*Dinner about* 6.—Fish, broiled or roasted, with meat, or meat alone. This is the best time for delicately boiled vegetables, such as spinach, or other green stuff, which may be varied according to the season. A great variety at a meal is bad, not only in itself but because it prevents change from day to day. If we do with few kinds of meat or fish it will be much easier to provide a change day by day than if we bring them all out every day as some people do. A change of food is most important, but variety of food at a meal is bad. An occasional dinner of fish, without meat, and even a purely vegetable dinner, if you can design a good one, is desirable. Stewed or fresh fruit is much better in your case than heavy milk puddings, which are forbidden.

Rules.—Some rules must be followed which are as necessary as the preceding. Take three meals a day at regular hours, about 8, about 1, and 5:30 to 6. Country people, and those not tormented by rushing about town all day, had better dine in the middle of the day, but you must dine at night when your day's work is over, or ought to be. Sip a glass of pure spring-water on waking and half a glass when going to bed. Let it be the best you can get, and at any temperature you desire; in summer the temperature of the air is the most agreeable. You must not drink at meals; in the end you will find this the most agreeable way. If thirsty, drink quite between meals or half an hour before meals. Each day sponge with water, and give a vigorous general friction with a towel. Use hair gloves all over the body twice a day. No food whatever should be taken between meals—no tea, cake, or anything of the kind. A cure will be hopeless unless you sleep well. Should anything prevent you sleeping let nothing prevent you from having seven hours in bed, whether you sleep or not. Take exercise between the meals, and after the latest meal of the day walk not less than half an hour. Do not work after dinner. Masticate the food well and slowly. Always keep the window open in your bed and working rooms, but the body well clothed or at night well covered up. Never overload the stomach; never eat to satiety. Sedentary people must take exercise morning or evening or at some time, or they will get ill.—*Farm and Home* (*English*).

